# Human-scale lung regeneration based on decellularized matrix scaffolds as a biologic platform

**DOI:** 10.1007/s00595-020-02000-y

**Published:** 2020-05-04

**Authors:** Keiji Ohata, Harald C. Ott

**Affiliations:** 1 Center for Regenerative Medicine, Massachusetts General Hospital, Harvard Medical School, 185 Cambridge Street, CPZN 4800, Boston, MA 02114 USA; 2 Division of Thoracic Surgery, Department of Surgery, Massachusetts General Hospital, Harvard Medical School, Boston, MA USA

**Keywords:** Lung regeneration, Extracellular matrix, Scaffold, Lung transplantation

## Abstract

Lung transplantation is currently the only curative treatment for patients with end-stage lung disease; however, donor organ shortage and the need for intense immunosuppression limit its broad clinical application. Bioartificial lungs created by combining native matrix scaffolds with patient-derived cells might overcome these problems. Decellularization involves stripping away cells while leaving behind the extracellular matrix scaffold. Cadaveric lungs are decellularized by detergent perfusion, and histologic examination confirms the absence of cellular components but the preservation of matrix proteins. The resulting lung scaffolds are recellularized in a bioreactor that provides biomimetic conditions, including vascular perfusion and liquid ventilation. Cell seeding, engraftment, and tissue maturation are achieved in whole-organ culture. Bioartificial lungs are transplantable, similarly to donor lungs, because the scaffolds preserve the vascular and airway architecture. In rat and porcine transplantation models, successful anastomoses of the vasculature and the airway were achieved, and gas exchange was evident after reperfusion. However, long-term function has not been achieved because of the immaturity of the vascular bed and distal lung epithelia. The goal of this strategy is to create patient-specific transplantable lungs using induced pluripotent stem cell (iPSC)-derived cells. The repopulation of decellularized scaffolds to create transplantable organs is one of possible future clinical applications of iPSCs.

## Clinical background

Currently, lung transplantation is the only curative option for patients with end-stage lung disease; however, its broad clinical application is limited by donor organ shortage, the need for immunosuppression, and chronic lung allograft dysfunction [1]. Recently, the discovery of induced pluripotent stem cells (iPSCs) has opened new opportunities in regenerative medicine [2, 3], and there has been increasing interest in translating stem cell therapy into clinical application. Despite ongoing progress in the field of regenerative medicine, the development of parenchymal organ engineering has slowed because of the high levels of architectural and functional complexity of vital organs. In the field of regenerative respiratory medicine, several research groups have reported successful differentiation of human stem cells toward pulmonary epithelial cells of various types, including both distal alveolar type II cells and proximal airway epithelia [4–11]. However, the successful functional engraftment of these cells into injured lungs has not yet been accomplished, and no optimal implantation strategies for the cells have been developed, despite their great therapeutic potential.

Our approach to these hurdles is to use whole-organ matrix scaffolds as a platform to bioengineer a functional organ. We build on the strategies of tissue and organ engineering to combine cells with biocompatible scaffolds to generate biologic devices to replace lost function [12, 13]. This review summarizes our achievements to date and discusses the role of lung matrix scaffolds in lung bioengineering.

## The role of extracellular matrix scaffolds as a biologic platform

The extracellular matrix (ECM) is a three-dimensional (3-D) molecular network that provides structural and biochemical support for embedded cells. ECM was initially considered simply as a space-filling component of tens of trillions of cells in the human body [14]. We know now that ECM provides essential extracellular environments for multiple types of cells and has a profound impact on cell growth, survival, and differentiation. ECM is first synthesized by embryonic cells during intrauterine development, and cell–ECM interactions are continuous, serving to influence gene expression and determine the cell behavior [15, 16]. Specifically, the interaction between the ECM and the cellular receptors regulates cell behavior and tissue morphogenesis. Among several important observations, the ECM of muscle was found to promote cell maturation of muscle progenitors as well as differentiate embryonic stem cells to maturing muscle tissue [17]. Likewise, fibronectin, a major component of the ECM, supports the attachment of rat alveolar type II epithelial cells [18]. In wound healing, the ECM promotes the migration and proliferation of progenitor cells and accelerates wound closure [19].

The main components of the ECM include collagens, glycoproteins (such as laminin and fibronectin), proteoglycans, and others, including elastin and hyaluronic acid. Collagen is the major structural component of the ECM and type I collagen is the most abundant subtype detected in tissues. Type IV collagen is a key component of the basement membrane and is among the most important factors to establish and maintain the “air–blood barrier” in the lungs. Laminin and fibronectin are both essential for cell survival. Cells attached to these components receive signals that have immediate impact on their morphology, motility, and differentiation [20, 21]. Proteoglycans are a diverse group of core proteins linked to sulfated polysaccharides or glycosaminoglycans. They bind growth factors and cytokines and retain them within the matrix. Elastin contributes to the mechanical nature of the lung and is a key feature of tissue recoil, which is critical for ventilation. These biologic characteristics are widely applied for in vitro cell culture methods and pharmacotherapeutic fields [22, 23]. As such, a key challenge is to determine how to reproduce the 3-D matrix architecture and promote physiologic ECM-cell interactions and functional gas exchange in lung tissue.

## Decellularization of cadaveric lungs

### Strategy for lung decellularization

In 2008, our group reported the development of decellularized whole-organ scaffolds as platforms for regenerative medicine [13]. Lung decellularization can be accomplished by several techniques, all involving perfusion throughout the native vascular system [24–28]. Decellularization is the process via which all cellular components from cadaveric organs are removed, resulting in isolated natural ECM scaffolds. The end points of adequate decellularization include removing all cellular material and minimizing loss of ECM composition, while at the same time minimizing disruption of the ultrastructure. Different types and combinations of detergents have been successfully employed toward this goal, including sodium dodecyl sulfate (SDS, anionic detergent), sodium deoxycholate (anionic), Triton X-100 (nonionic), Tween (nonionic), and 3-[(3-cholamidopropyl) dimethyl ammonio]-1-propanesulfonate (CHAPS, zwitterionic detergent). Tsuchiya et al. reported that CHAPS solution at low pH suppressed loss of glycosaminoglycans and elastin, but was not effective at removing all cellular DNA [29]. Dextrose pretreatment might be useful for increasing protein stability and reducing collagen loss during decellularization [30], and additional airway perfusion with detergent solution is an option for more effective decellularization [31]. An attempt to generate vascularized scaffold grafts was performed by a selective cell removal process that targeted only airway cells while keeping vascular cells intact [32]. Importantly, this procedure is applicable to any cadaveric solid organ, including the heart [25, 33], liver [34], kidney [35], pancreas [36], small intestine [37], and limbs [38].

**Fig. 1:**
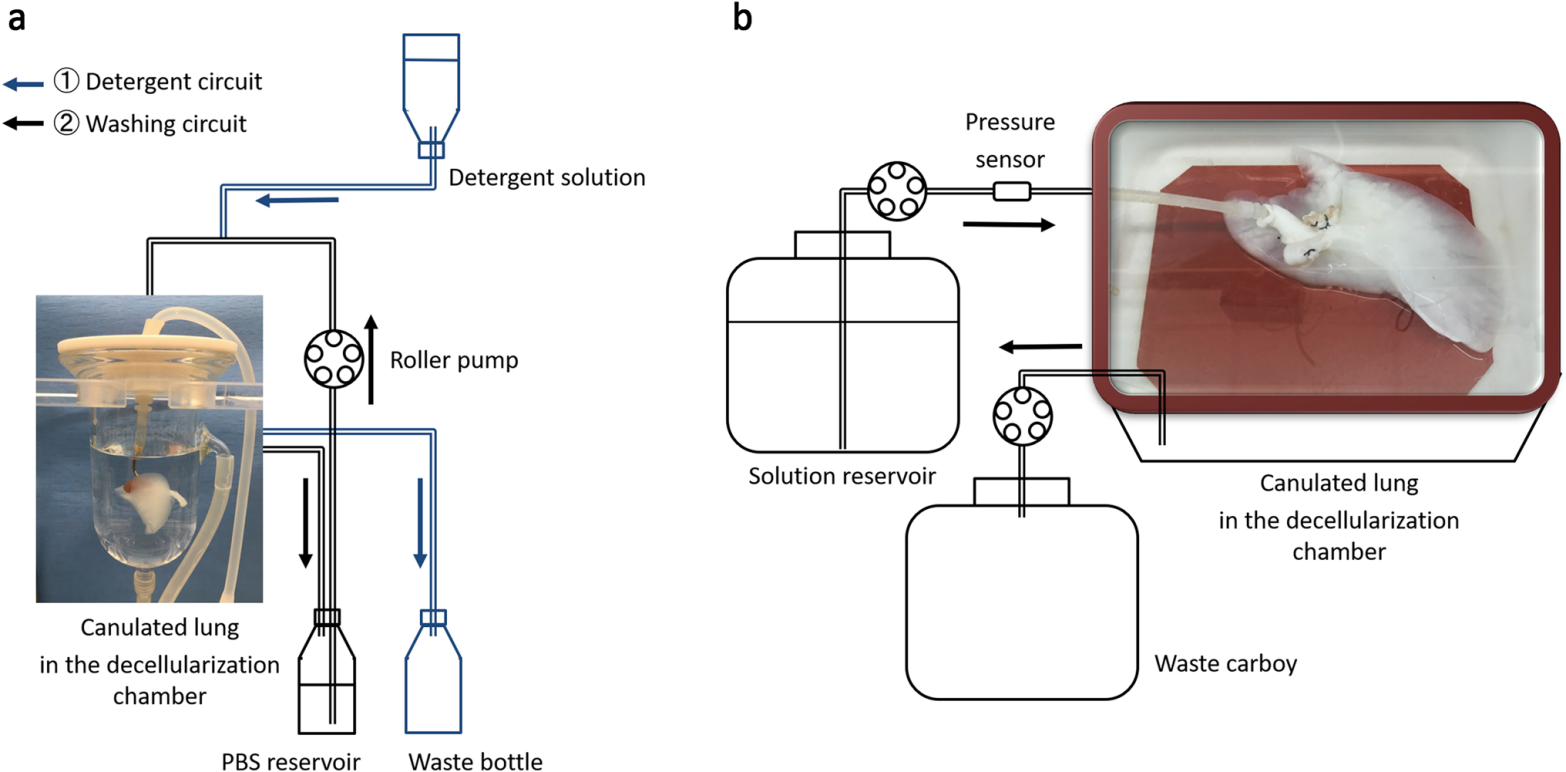
The decellularization systems for rat lungs (**a**) and larger lungs from swine, monkeys, and humans (**b**). **a** Lungs procured from heparinized donors are sequentially perfused with sodium dodecyl sulfate (SDS) solution, distilled water, and Triton X-100 through a cannula placed in the pulmonary artery under a gravitational pressure of 80 mmHg. Detergent solution is infused into the lung through pulmonary arterial canula under a gravitational pressure of 80 mmHg and the effluent is drained to the waste bottle (blue arrow). After detergent infusion, phosphate buffered saline (PBS) circulates the circuit by the roller pump and wash the lung (black arrow). **b** The porcine left lung is placed in the custom-made decellularization chamber and perfused with detergent solutions and PBS through the pulmonary artery. The flow rate is controlled by the roller pump to maintain a perfusion pressure below 15 mmHg. The effluent is drained to the waste carboy

**Fig. 2:**
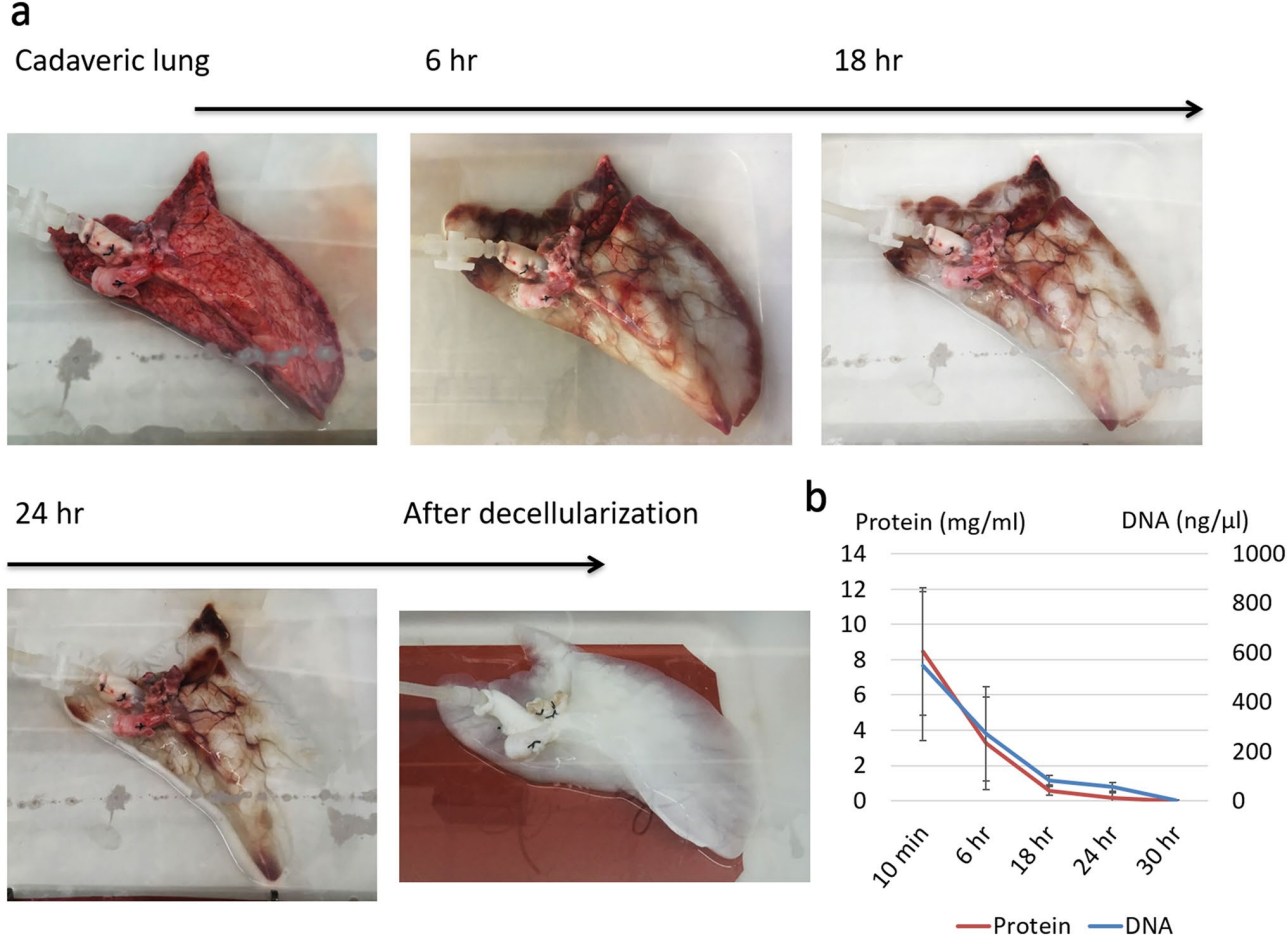
**a** The gross appearance of changes in the porcine lung during decellularization process. The lung lost opacity as the cellular material was washed out. **b** DNA and protein content in the effluent gradually decreased in association with the change in lung appearance

**Fig. 3:**
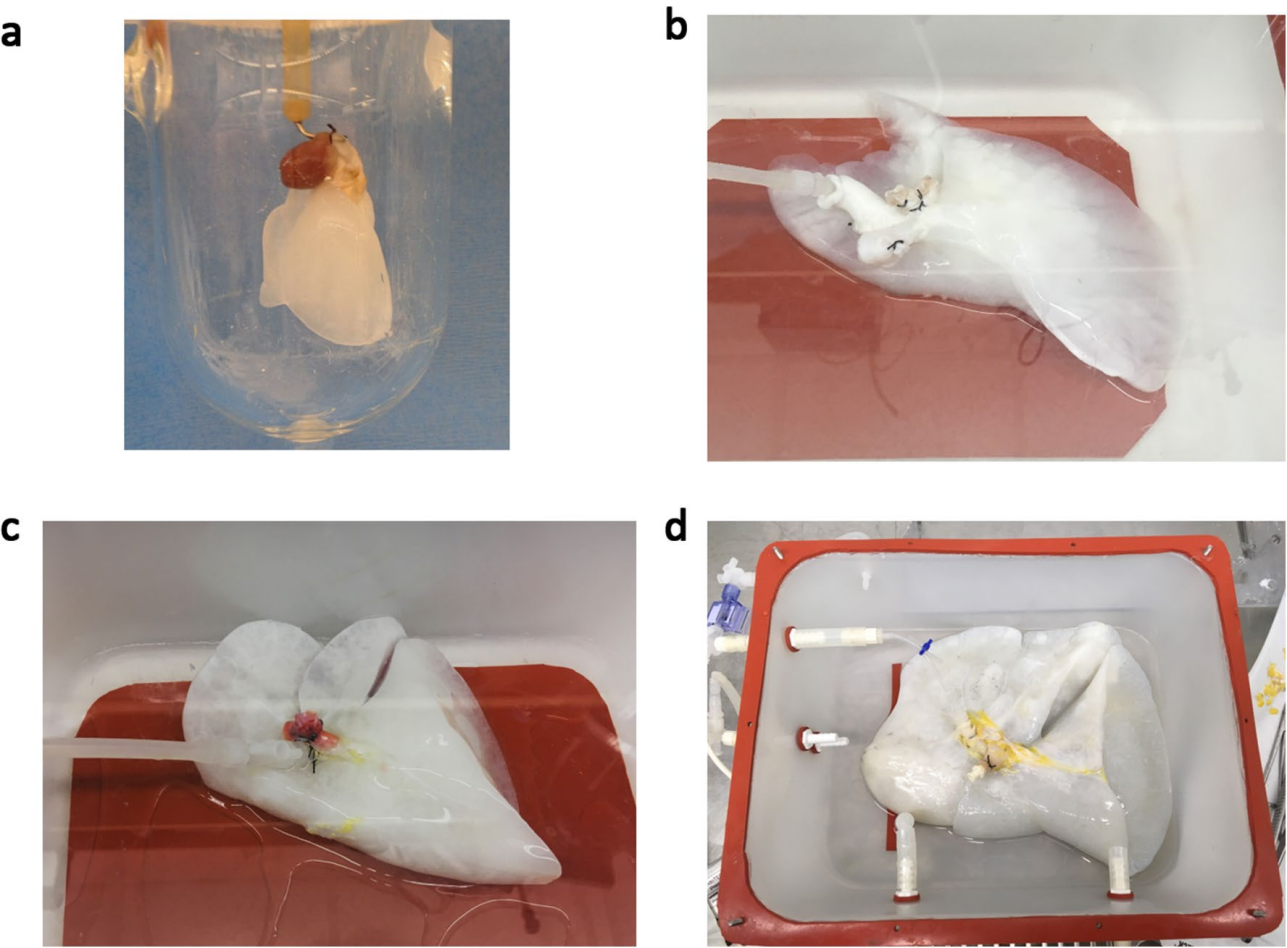
Gross appearance of decellularized lungs procured from **a** rat, **b** swine, **c** macaque, and **d** human

Acellular lung scaffolds were obtained via perfusion-based decellularization of cadaveric lungs. Detergent perfusion effectively removed cellular material from the tissue by solubilizing cell membranes. Table 1 and Fig. 1 include our decellularization protocols and systems used for rat lungs (Fig. 1a) and also for larger lungs from swine, monkeys, and humans (Fig. 1b). Lungs procured from heparinized donors were sequentially perfused with SDS solution, distilled water, Triton X-100 solution, and phosphate-buffered saline through a cannula placed in the pulmonary artery within custom-made decellularization chambers. The perfusion with the SDS solution removed cellular components and the 1% Triton X-100 wash step facilitated the removal of residual SDS within the tissue. Vascular resistance increased abruptly on the initiation of SDS perfusion because of the high viscosity of the solubilized cellular content, and gradually decreased as the cellular content decreased; as such, constant and controlled low-pressure perfusion provided optimal results. We maintained a perfusion pressure below 15 mmHg during the decellularization process to prevent breakage of the very friable capillary basement membranes. Decellularization of human and large animal lungs required higher concentrations of detergent solution and longer perfusion times than those used in rodents. We found that the lungs lost opacity as the cellular material was washed out (Fig. 2a). DNA and protein content of the decellularization effluent also decreases gradually in association with the change in lung appearance (Fig. 2b) [39]. We also successfully scaled up the decellularization process to be achieved for lungs from pigs, monkeys, and humans (Fig. 3).

**Table 1:** Decellularization protocols for rat lungs and larger lungs from swine, monkeys, and humans

Decellularization protocol for rat lungs				
	Solution	Concentration	Perfusion flow	Duration
1	SDS	0.1% (wt/vol)	Gravity pressure of 80 mmHg	120 min
2	Distilled water		Gravity pressure of 80 mmHg	15 min
3	Triton X-100	1% (vol/vol)	Gravity pressure of 80 mmHg	10 min
4	PBS		Roller pump flow of 4 ml/min	2–3 days

Decellularization protocol for lungs from swine, monkeys, and humans				
	Solution	Concentration	Perfusion flow	Duration
1	SDS	0.5% (wt/vol)	Constant pressure of 10–15 mmHg with roller pump	30 h
2	Distilled water		Constant pressure of 10–15 mmHg with roller pump	12 h
3	Triton X-100	1% (vol/vol)	Constant pressure of 10–15 mmHg with roller pump	12 h
4	PBS		Constant pressure of 0–15 mmHg with roller pump	2–3 days

*SDS* sodium dodecyl sulfate, *PBS* phosphate buffered saline

### Key features of decellularized lung scaffolds as a biologic platform for lung regeneration

**Fig. 4:**
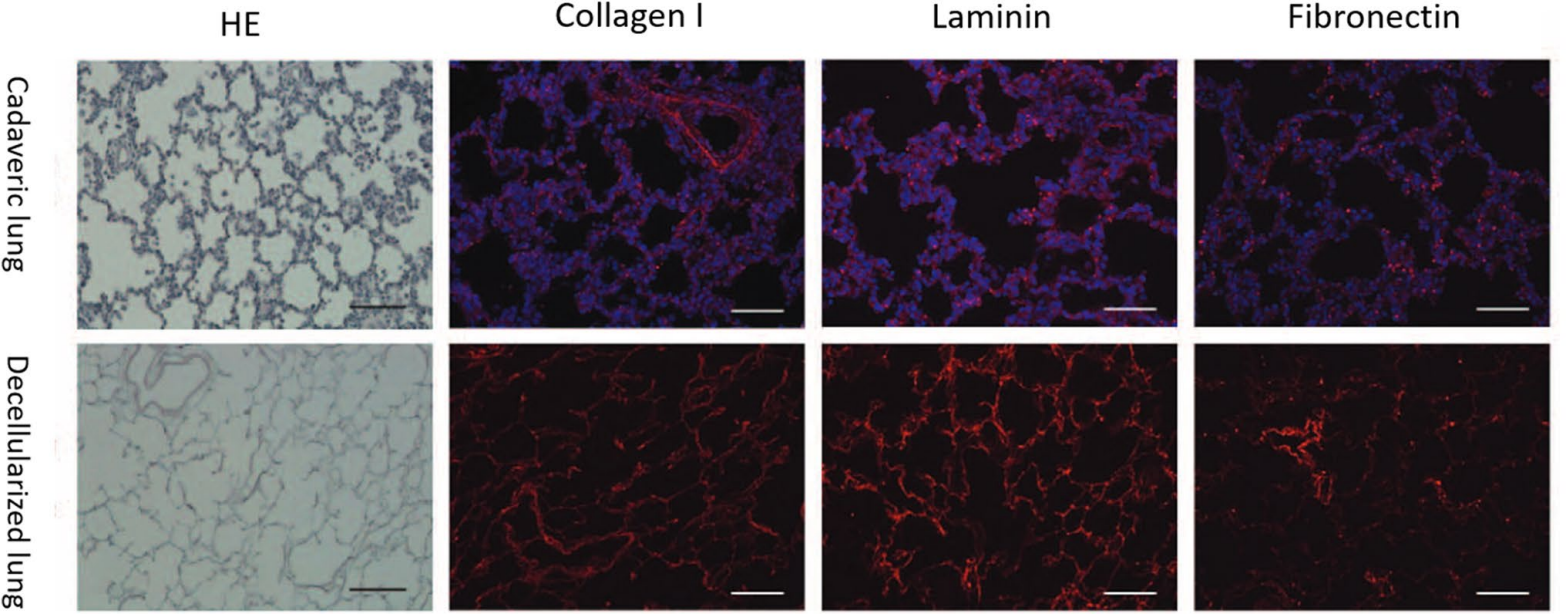
Hematoxylin and eosin (HE) staining and immunofluorescent (IF) staining of the porcine cadaveric lung and decellularized lung scaffold. HE staining showed that decellularized scaffolds preserve the alveolar structure of the lungs. IF staining revealed the complete absence of cellular nuclei but the preservation of and matrix proteins including type I collagen, laminin, and fibronectin in the decellularized lung. Scale bars 100 µm

Decellularized native scaffolds preserve both the micro- and macroarchitecture of the native lung and retain much of the original ECM composition. One of the difficulties inherent in creating any parenchymal organ is the necessary reproduction of its critical and complex 3-D architecture. The minimum functional unit of the lung is the highly specialized “air–blood barrier” between the alveoli and capillaries. Gas exchange requires close interaction between interalveolar air and blood circulation across the alveolar epithelium, basement membrane, and vascular endothelium. Reproducing this ultrathin barrier using conventional manufacturing techniques has not been accomplished to date. Decellularized lung scaffolds preserve the 3-D microarchitecture of the lungs and enable the alveolar epithelium and vascular endothelium to form monolayers with a basement membrane and intact barrier function and basement (Fig. 4) [39]. Furthermore, alveoli and capillaries are connected within hierarchical conducting systems, the bronchial tree, and the vascular network, respectively, which facilitates near-immediate contact between ambient air and circulating blood. Preservation of the intact vascular network and bronchial tree allows direct connection between the transplant and the vasculature and airway, either orthotopically or heterotopically. The hierarchical branching system also enlarges the alveolar surface area and maximizes the efficiency of gas exchange. Even with advanced manufacturing technology, including 3-D printing, the complex architecture of the lung has not been reproduced successfully; thus, acellular lung scaffolds remain the most promising approach.

Decellularized native scaffolds retain some of the original ECM composition and can provide cells with site-specific support. Histologic analysis of a porcine lung acellular scaffold showed no cellular nuclei, but preservation of type I collagen, laminin, and fibronectin in the decellularized lung (Fig. 4) [39], thereby providing the unique and critical organ-specific environment for cell growth and maturation. Some ECM components are inevitably lost in any decellularization protocol, and quantitative measurements of ECM components remaining after perfusion have revealed reductions in elastin and sulfated glycosaminoglycans [29, 39]. As an intricately interwoven network of collagen and elastin fibers provides important mechanical support for respiration, the decrease in elastin may contribute to extensive problems with compliance and lack of recoil in the decellularized lungs. To obtain a more suitable platform for tissue engineering, methods for enhancement of acellular scaffolds are in development. In the cardiovascular field, autologous pericardium treated with a glutaraldehyde solution has been used for aortic valve reconstruction [40]. Glutaraldehyde strengthens ECM by cross-linking collagen, which might be useful for generating whole-organ matrix scaffolds. From a functional aspect, Chen et al. reported that using a decellularized bone scaffold coated with stromal cell-derived factor-1α improved endogenous stem cell recruitment and enhanced osteogenesis in a rabbit model [41]. We analyzed compositional differences between human neonatal and adult lung ECM and found that fibrillin-2 and tenascin-C were both expressed at higher levels in the neonatal ECM. Accordingly, supplementation with fibrillin-2 and tenascin-C resulted in increased epithelial cell proliferation in both in vitro and ex vivo 3-D scaffold cultures [42].

### Xeno-organs as scaffolds for human tissue

Declined human donor lungs are the most logical and immediate candidates for decellularization and clinical application, as these have already been used extensively for experimental purposes [43, 44]. However, donor lungs are an inherently heterogeneous source and have experienced varying degrees of injury from the circumstances of death and donation. For example, re-seeded cells did not survive beyond 1 week in decellularized emphysematous human lungs; however, this scaffold may lack the essential 3-D ECM architecture to support cell growth [45]. Likewise, matrix scaffolds created from the lungs of patients with idiopathic pulmonary fibrosis displayed significant compositional differences from the normal lung, and the fibrotic matrices promoted myofibroblast differentiation [45]. The relationship between lung damage and matrix functionality needs to be further investigated.

Scaffolds generated from pig lungs might be a feasible alternative because of the striking anatomic similarities between porcine and human organs. An animal source for these scaffolds would facilitate precise quality control. Porcine scaffolds could be a by-product of the food industry, whereby we could take advantage of a nearly unlimited and highly consistent supply of donor tissue. ECM components are highly conserved across species, and xenogeneic scaffold devices have already been used in clinical practice for a variety of reconstructive procedures, including heart valve replacement and wound care [46]. A major factor underlying the hyperacute rejection of pig organ transplants in primates is an acute reaction to the galactose-α-1,3-galactose (Gal)-epitope. However, the Gal-epitope in porcine ECM scaffolds did not elicit a host response or tissue remodeling because of the relatively small amount of antigen present in the tissue [47]. Furthermore, genetically modified α1,3-galactosyltransferase gene-deleted pigs are now available. The absence of the Gal-epitope from porcine lung tissue delayed immune cell infiltration and reduced the chronic T cell-mediated reaction against decellularized materials following reimplantation [48]. As such, removing the Gal-epitope may be an effective strategy for extending the life of clinical-grade decellularized scaffolds. Porcine organs might be an option for whole-organ scaffolds in future clinical application.

## Recellularization of lung matrix scaffolds

### Cell types for recellularization and cell infusion strategy

Optimization of recellularization and maturation protocols is a current challenge in bioengineering clinically useful acellular scaffolds. Repopulation of organ scaffolds requires appropriate cell population, which will provide high-level organ-specific function. The first step in organ scaffold recellularization is a cell infusion procedure in which appropriate cell types are placed at relevant sites in the scaffolds. Techniques to effectively deliver specific cell populations into the correct niches throughout the lung scaffold are being developed.

The biggest challenge in creating bioartificial lungs from scaffolds is the need to reproduce functional and stable vascular networks that play an essential role in the gas exchange function, nutrient supply, and waste transport from the lungs: vascular endothelial cells are the primary population contributing to these functions. For vascular cell re-seeding, cell suspensions are infused simultaneously through the pulmonary artery and pulmonary vein under a gravitational pressure of 80 mmHg. Gravity-driven cell infusion contributed to the even distribution of cells within the vasculature and supported cell survival. In contrast, seeding driven by a roller pump device led to increased cell death [49]. The use of low-concentration and high-volume cell suspensions resulted in an even distribution of cells throughout the lung scaffolds. After cell infusion, a static period, followed by simultaneous arterial and venous perfusion, facilitates cell attachment to the matrix. Incomplete endothelial cell coverage within the vasculature promotes thrombosis secondary to platelet activation and adhesion to the ECM or alveolar hemorrhage. Exposed ECM proteins in the scaffold may also invoke aberrant immune responses upon implantation. Collagen type V has been identified as a critical factor contributing to chronic lung allograft dysfunction [50]. A variety of cells have been used to repopulate the pulmonary vasculature, including primary cells from rat lung microvessels [24], human umbilical vein endothelial cells [39], and iPSC-derived vascular endothelial cells [44]. A number of cells that are sufficiently able to obtain cell–cell interactions and paracrine activities within the scaffold should be included in the infusions.

The parenchymal cells responsible for the specific functions of the organ are also necessary. To date, different airway epithelial cells, including a heterogeneous mixture of fetal or neonatal rat lung cells [24, 25], carcinomatous human alveolar epithelial cells [25], human airway basal stem cells [39, 51], and iPSC-derived epithelial cells [7, 52, 53], have been used to re-epithelialize the airways of lung scaffolds. In an airway re-seeding procedure, the epithelial cell suspension is infused via a bronchus or the trachea. The dead-ended architecture of the bronchial tree makes it difficult to achieve universal and even delivery to all sites. Some approaches that have already been used for intratracheal cell transport and delivery might be adopted for acellular scaffolds [54]. An improved understanding of intratracheal cell transport dynamics and development of suitable cell monitoring techniques are both needed.

In addition to the cells that reconstitute the epithelium and endothelium, other cell lineages need to be incorporated, including pericytes and smooth muscle cells [44, 55]. Likewise, fibroblasts enhance the functional phenotype of the parenchymal cells and contribute to the organization of the tissue architecture. The introduction of undifferentiated lung stem cells into these microenvironmental niches might contribute to tissue homeostasis after implantation for long-term success.

### *Development of bioreactor and *ex vivo* organ culture*

The lung scaffolds are recellularized in a bioreactor that facilitates cell delivery and perfusion with culture medium via the pulmonary artery, vein, and airway. After the cell attachment, the cells on the ECM scaffold are maintained in an organ-specific biomimetic environment. Developing specialized bioreactors that support cell growth and maturation within the bioartificial lungs is another important challenge in this field. Some researchers have designed bioreactors that promote nutrient, waste, gas, and cytokine exchange [56, 57].

**Fig. 5:**
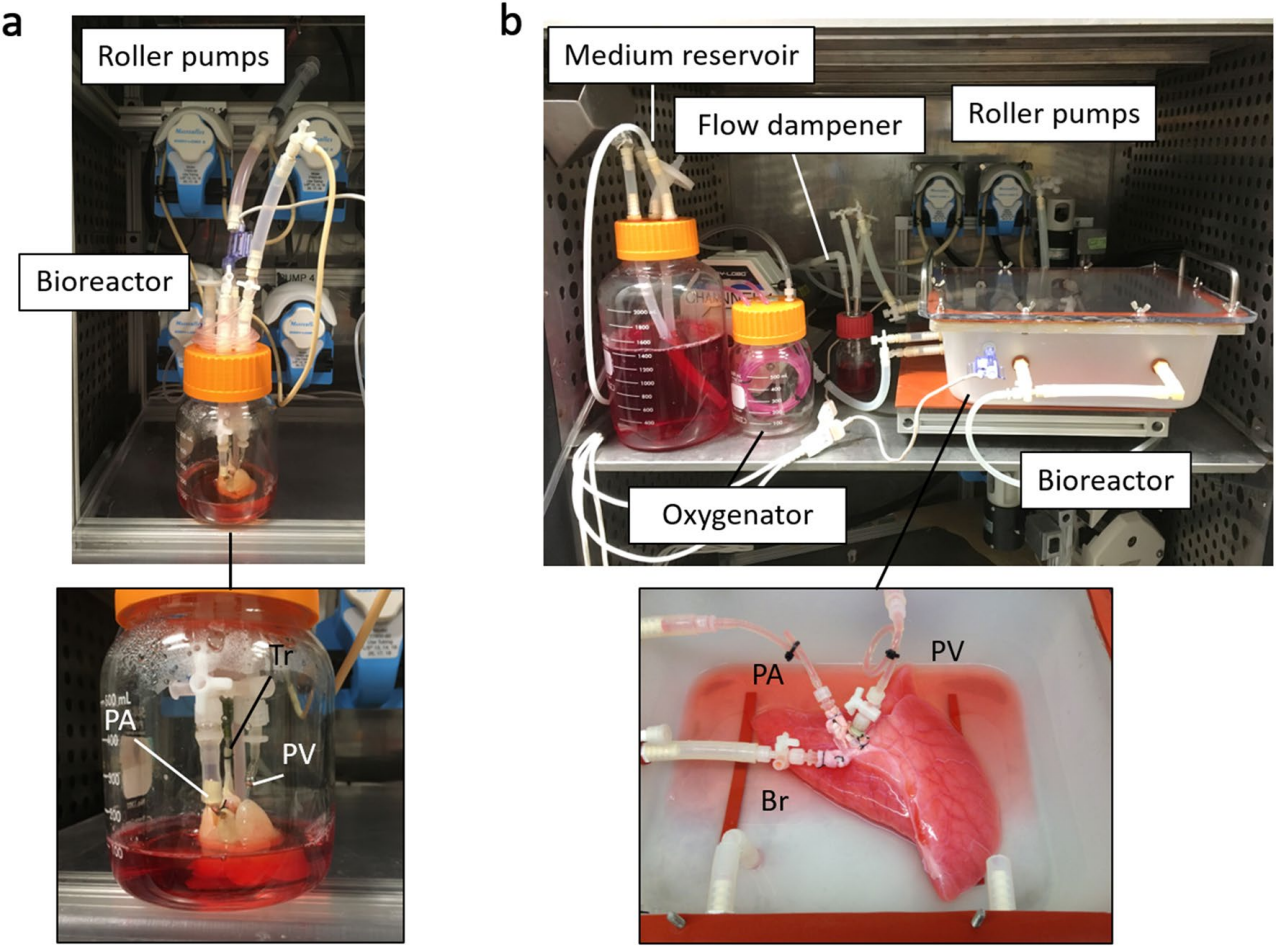
Our recellularization systems for rat- **a** and human-sized **b** lungs. Cell seeding, engraftment, and tissue maturation are achieved in the bioreactor, which provides biomimetic conditions, including vascular perfusion and liquid ventilation by roller pumps. An air-cushioned flow dampener facilitates smooth vascular perfusion that minimizes matrix damage. *PA* pulmonary artery, *PV* pulmonary vein, *Tr* trachea, *Br* left bronchus

Figure 5 shows our recellularization systems for rat- and human-sized lungs. Whole-organ culture systems are based on extracorporeal organ perfusion devices that drive culture medium through the graft. The lung bioreactor circulates perfusate from a reservoir through the lungs via the pulmonary artery. Perfusion culture with a sufficient flow rate and pulmonary arterial pressure can result in full recellularization of the lung vasculature and promote the delivery of oxygen and nutrition due to the relatively low vascular resistance, a notably crucial feature particularly in human-sized lung scaffolds. Smooth anterograde perfusion that minimizes matrix damage is facilitated by a roller pump with an air-cushioned flow dampener. Mechanical stimulation is a key factor that enhances cell growth and organ maturation in organ culture. The flow of culture medium through vascular scaffolds in the bioreactor produces shear stress as a result of hemodynamic forces. This biophysical stimulus promotes homing, paracrine effects, and differentiation of endothelial progenitor cells. An additional oscillation flow might be an option to enhance these beneficial effects. In more recent recellularization strategies, we employed open vein circulation, which includes a venous pressure of 0 mmHg against the hilum. Controlled positive venous pressure may contribute to more effective lung recellularization. In a study of ex vivo lung perfusion with pig cadaveric lungs, a left atrial pressure of 5 mmHg led to reduced edema and superior lung physiology [58].

Airway perfusion is another option in lung organ culture. The movement of medium in and out of the airways is driven by negative pressure in the bioreactor chamber and the addition of cyclic stretch may provide crucial developmental signals [59]. Petersen et al. reported that liquid ventilation promoted cell survival and maintained airway structure in cadaveric rat lung organ culture [60]. Alveolar dilation recruits capillary vessels and may also have a positive impact on the recellularization of microcapillaries. Previous lung organ culture successfully maintained recellularized scaffolds for several days and up to as long as 1 month [30, 39]. Noninvasive developmental strategies for real-time monitoring of recellularization are needed [61].

### Induced pluripotent stem cells as a source for lung recellularization

As we move toward the most clinically relevant model, ideal cell sources for recellularization must be identified and tested. This will most likely involve iPSC-derived cells. Yamanaka et al. successfully reprogrammed mouse and human fibroblasts to generate pluripotent stem cells [2, 3]. Repopulating scaffolds with donor-derived cells differentiated from iPSCs would facilitate personalized transplantation medicine and reduce the need for immunosuppressive therapy. The proliferating capacity of iPSCs suggests that it will not be difficult to obtain appropriately differentiated cells in sufficient numbers to regenerate organ function. Autologous primary cells isolated from harvested lung tissue are insufficiently proliferative and do not typically expand to the numbers required for rebuilding on our scaffolds; as such, they are less likely candidates for clinical application. In addition, lung cell collection with the intent to repopulate is not an option for patients with end-stage lung disease. The stem cell-specific microenvironments, also known as “niches” [62, 63], which serve to maintain stem cell renewal and differentiation have been described in a number of different organs. ECM proteins are among the most important components of this niche.

Cells would ideally be derived from nonimmunogenic sources, but allogenic cells might serve as candidates. Kyoto University currently maintains recorded stocks of iPSCs to be used for regenerative medicine and research. These stocks include iPSCs induced from healthy donors with defined human leukocyte antigen (HLA) profiles. Xu et al. reported on the use of CRISPR-Cas9-based HLA editing to create the immunocompatible iPSCs [64]. This will undoubtedly become a powerful source of iPSCs for both research and for clinical practice.

## Transplantation of bioartificial lungs

The primary outcome of lung bioengineering studies is in vivo gas exchange. As the lung scaffolds preserve the vascular and airway architecture from alveoli to the hilum, the bioartificial lungs should be transplantable in a fashion similar to that for cadaveric donor lungs. Initially, researchers from Yale University and our group demonstrated the feasibility of recellularized bioartificial lungs in a rat transplantation model [24, 25]. Petersen et al. repopulated acellular rat lung scaffolds with mixed populations of neonatal rat lung epithelial cells and microvascular endothelial cells [60]. We recellularized our scaffolds with carcinomatous human alveolar epithelial cells or rat fetal lung cells combined with endothelial cells. In both studies, the recellularized lungs were transplanted orthotopically into recipient rats, which resulted in partial restoration of respiratory function. After optimization, we created bioartificial rat lungs that were recellularized with rat fetal pneumocytes and human primary vascular cells. These grafts maintained respiratory function through the recipient’s airway for up to 7 days after implantation [65].

Scaling up from a small animal to a clinically relevant large animal model is a critical step toward human clinical application. Toward this end, we created human-sized bioartificial lung grafts by repopulating porcine lung scaffolds with human umbilical vascular endothelial cells and human basal airway stem cells. These grafts were transplanted heterotopically with the pulmonary artery and vein anastomosed with the pulmonary trunk and left atrial appendage, respectively. The graft withstood physiologic pulmonary blood flow and performed gas exchange for 1 h after surgical implantation [39]. We also created bioartificial lungs and performed orthotopic transplantation to assess feasibility and in vivo function using a porcine 24-h survival model. Technically successful orthotopic anastomoses of the vasculature and the airway were achieved, and perfusion and ventilation of the lung grafts were confirmed. Gas exchange was evident immediately after transplantation. Unfortunately, the graft lacked sufficient barrier function and an increase in thrombus formation led to graft failure within 24 h. Further progress toward optimizing recellularization and maturation of the grafts will be necessary to improve their sustainability and functionality. Nichols et al. recellularized porcine lung scaffolds with autologous cells from porcine recipients and transplanted them without vascular anastomoses [30]. In this study, the bioartificial lungs survived in the recipient thorax accompanied by further cell expansion and vascular tissue development after implantation.

## Application of organ scaffolds to other fields

Decellularized organ scaffolds also hold promise as a tool for modeling human disease. Lung scaffolds have been used to culture lung cancer cells in a biomimetic 3-D environment, presenting unique aspects of the biology of this disease [66, 67]. Mishra et al. reported that cancer cells in the scaffold produced matrix metalloproteinases that were known products of the cells in situ but were not detected in 2-D culture [68]. Lung scaffolds will also enable us to monitor other cancer cell characteristics under biomimetic conditions including proliferation and the relative sensitivity to chemotherapeutic agents [69].

The contributions of acellular scaffolds to decisions relating to the cell fate of iPSCs are important but incompletely understood. For example, the attachment of embryonic stem cell-derived lung epithelial cells to ECM proteins resulted in enhanced surfactant protein-C expression [70]. Human iPSC-derived lung organoids improved the cellular differentiation, and the resulting airway-like structures were similar to the human adult lung [63]. Modeling of human lung development, lung disease, and screening for effective drug therapies might all be improved with the future use of iPSC-derived cells for this purpose.

## Conclusions

Acellular lung scaffolds from small and large animals, which have preserved the original vascular network, bronchial tree, and most of the ECM composition, were generated via perfusion of the pulmonary artery with detergent solutions. The resulting lung scaffolds were recellularized in bioreactors and successful cell growth was achieved with lung perfusion culture. The resulting bioartificial lungs with the vascular and airway architectures preserved would be used in transplant studies. In rat and porcine transplantation models, the bioartificial lungs that were transplanted promoted gas exchange after implantation, although graft failure resulted from insufficient vascular barrier function and increased thrombogenicity. As such, further progress in optimizing the recellularization and the maturation of the grafts will be necessary to improve the sustainability and the functionality of the grafts. While early in its development, organ engineering provides the unique potential to promote personalized treatment options based on recellularization with patient-derived iPSCs.
